# Supplementary material for: The plastome sequence of Bactris gasipaes and evolutionary analysis in tribe Cocoseae (Arecaceae)
Source: PLoS One. 2021 Aug 24;16(8):e0256373. doi: 10.1371/journal.pone.0256373 (PMC8384209; doi:10.1371/journal.pone.0256373)
Supplement: S1 Table — (DOC) [file pone.0256373.s001.doc]

**S1 Table.** GenBank accession numbers of the nucleotide sequences used in our analysis.

| **Subtribe** | **Species** | **Genbank Accession** | | |
| --- | --- | --- | --- | --- |
|  |  | **PRK** | **RPB2** | **Plastome** |
| Elaeidinae | *Elaeis guineensis* | AY601219 | HQ265661 | NC_017602 |
| Bactridinae | *Astrocaryum aculeatum* | JQ821944 | JQ821977 | MH537788 |
|  | *Astrocaryum murumuru* | HQ265590 | HQ265637 | MH537787 |
|  | *Bactris gasipaes* | KP218842 | HQ265650 | XXXXXX |
|  | *Acrocomia aculeata* | HQ265574 | HQ265620 | NC_037084 |
| Attaleinae | *Cocos nucifera* | HQ265608 | EF491150 | NC_022417 |
|  | *Butia eriospatha* | - | - | MN329806 |
|  | *Butia capitata* | AY601252 | EF491157 | - |
|  | *Syagrus coronata* | - | - | NC_029241 |
|  | *Syagrus smithii* | AY601263 | HQ265666 | - |
