# Supplementary figures and images for: The plastome sequence of Bactris gasipaes and evolutionary analysis in tribe Cocoseae (Arecaceae)
Source: PLoS One. 2021 Aug 24;16(8):e0256373. doi: 10.1371/journal.pone.0256373 (PMC8384209; doi:10.1371/journal.pone.0256373)

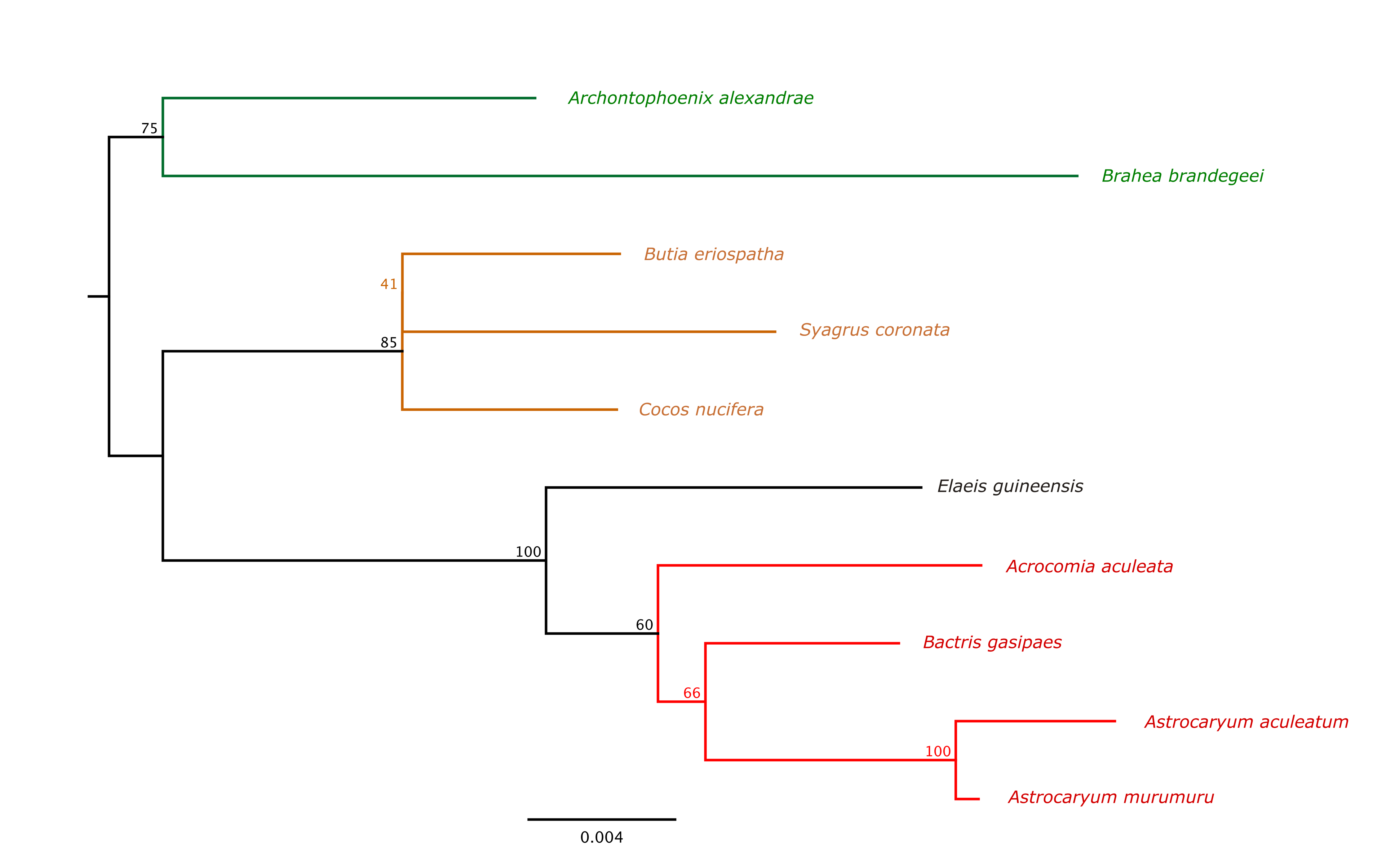

Supplement: S1 Fig — The numbers above the branches are maximum likelihood bootstrap values (1000 replicates). (TIF) [file pone.0256373.s002.tif]
