# Supplementary material for: The plastome sequence of Bactris gasipaes and evolutionary analysis in tribe Cocoseae (Arecaceae)
Source: PLoS One. 2021 Aug 24;16(8):e0256373. doi: 10.1371/journal.pone.0256373 (PMC8384209; doi:10.1371/journal.pone.0256373)
Supplement: Attachment — Submitted filename: The plastome sequence of Bactris gasipaes and evolutionary analysis in tribe 24052021.docx [file pone.0256373.s003.docx]

The plastome sequence of Bactris gasipaes and evolutionary analysis in tribe

Cocoseae (Arecaceae)

--Manuscript Draft-- PONE-D-21-11791

Ibrahim S. Al-Mssallem

**Abstract:** Written straightforwardly and clearly. The length of the abstract abides with the Journal guidelines, which include 6 points:

1-The family Arecaceae is distributed throughout tropical and subtropical regions of the world. Among the five subfamilies, Arecoideae is the most species-rich and still contains some ambiguous inter-generic

relationships.

2- The hypervariable regions of plastid genomes (plastomes) are interesting tools to clarify unresolved phylogenetic relationships.

3- sequence and characterized the plastome of Bactris gasipaes (Bactridinae) and compared it with eight species from the three Cocoseae sub-tribes (Attaleinae, Bactridinae, and Elaeidinae) to perform comparative analysis and to identify hypervariable regions.

4- The Bactris gasipaes plastome has 156,646

bp, with 113 unique genes. Four genes have an alternative start codon (cemA, rps19, rpl2, and ndhD). Plastomes are highly conserved within tribe Cocoseae: 97.3 % identity, length variation of ~2 kb, and a single ~4.5 kb inversion in Astrocaryum plastomes.

5- The LSC/IR and IR/SSC junctions vary among the subtribes: in Bactridinae and Elaeidinae, the rps19 gene is completely contained in the IR region subtribe Attaleinae the rps19 gene is only partially contained in the IRs. The hypervariable regions selected according to sequence variation (SV%) and frequency of parsimony informative sites(PIS%) revealed plastome regions with great potential for molecular analysis.

 6-The ten regions with the greatest SV% showed higher variation than the plastid molecular markers commonly used for phylogenetic analysis in palms. The phylogenetic trees based on the plastomes and the hypervariable regions (SV%) datasets had well-resolved relationships, consistent topologies within tribe Cocoseae, and confirm the monophyly of the subtribes Bactridinae and Attaleinae.

**Introduction**; It covers the objectives of the study in a very acceptable manner, which include:

Arecaceae family taxonomy(genera and species) and distribution and the most recent taxonomic review in Arecaceae,

Plastid genomes (plastomes) highlight the. Hypervariable regions in plastomes structural rearrangements, loss of genes, introns, and heterogeneous nucleotide substitution rates in protein-coding genes among their plastomes [5,6]. The Mutation rate in the plastome. In addition, in phylogenetic analysis, these hypervariable regions may also be robust molecular markers for analysing population genetic structure, including that among wild and domesticated populations. The importance of Arecaceae includes species of great ecological, economical (high-value products for industry and nutritional. The literature review is more than enough and covers more than 54 articles between 1992-2020

**Material and Methods: Presented and applied in the** conventional and standard approaches, which include:

Taxon sampling, Molecular, Genomics and Bioinformatics. Libraries were prepared with Nextera XT DNA Library Preparation Kit (Illumina, San Diego, CA, USA) and sequenced in Illumina. Plastome assembly was performed using CLC Genomics Workbench v.8.0 (Qiagen, Germantown, MD, USA) software with a de novo strategy. Plastome annotation was performed using Geneious Prime® (Biomatters, Auckland, New Zealand). The final plastome sequence was deposited in GenBank: MW054718.

Plastome structural analysis in tribe Cocoseae: The comparative analysis to identify structural rearrangements in the plastomes of the Cocoseae species was carried out using eight species and finally, Identification of hypervariable regions and Phylogenetic inferences

**Result and discussion**

1-the average read length of 214 bp, which is relatively small compared to another study, e.g. Yang et al. 2010. unfortunately, no average depth of coverage presented?

2- The assembled plastome has a 21 bp gap in the IGS trnT-UGU/trnL-UAA. This gap is in an AT-rich region (sequence 22 bp upstream to 119 bp downstream is only 7.8% of GC-content) and is, therefore, difficult to sequence.

3- Bactris gasipaes plastome structure and data are very close to plastomes in angiosperm, e.g. Date palm: has the quadripartite structure, with a pair of inverted repeat (IRs=27,038 bp), a large single-copy region (LSC= 85,118 bp), and a small single-copy region (SSC=17,452 bp). resulting in a plastome with 156,646 bp. ( Date palm cp158462 bp)

4- Bactris gasipaes **plastome has low GC content, an average of 37.5% compared to the date palm, 62.77%.** Unfortunately, no data on SNP.

5- In the Bactris gasipaes plastome, 113 unique genes annotated genes, 79 of which are protein-coding genes, 30 tRNA genes, and 4 rRNA genes (Table 2). Duplicate genes in IRs include 8 tRNA genes, 4 rRNA genes, and 6 protein-coding genes (Table 2). Among the 113 genes, 15 genes contain one intron (6 tRNA genes and 9 protein-coding genes), and 3 genes contain two introns (clpP, ycf3, and rps12; Table 2). Among intron-containing genes, 12 are located in LSC (trnK-UUU, rps16, trnG-UCC, atpF, rpoC1, ycf3, trnL-UAA, trnV-UAC, clpP, petB, petD, rpl16), 1 in SSC (ndhA), 4 in IRs (rpl2, ndhB, trnI-GAU, trnAUGC), and rps12 is a trans-splicing gene with the first exon located in the LSC region and the second and third exons in the IRs**.( most of the data observed in cp of Date palm)**

5- the cemA gene exhibited an alternative start codon, as reported in subtribes Attaleinae and Elaeidinae, contrasting with the Bactridinae species sequenced so far (Astrocaryum aculeatum, Astrocaryum murumuru, and Acrocomia aculeata). Three other genes have alternative initiation codons, rps19 (GTG), rpl2 (ACG), ndhD (ATC)**. (some data observed in cp of Date palm).**

**II- *Comparative analysis in tribe Cocoseae***

1- Plastomes are highly conserved within the tribe Cocoseae. Species from subtribes Bactridinae and Elaeidinae have a plastome ~2 kb larger than the subtribe Attaleinae. This difference in length between the subtribes is mainly in the IRs and LSC regions (Table 3). The plastomes from Cocoseae species ranged from 154.048 bp (Butia eriospatha) to 156.937 bp (Elaeis guineenses).

2- The progressive alignment among species from tribe Cocoseae shows evidence for three Locally Collinear Blocks (LCBs) (Fig. 1). These three LCBs result from the 4.5 kb inversion present in the plastomes of Astrocaryum murumuru and Astrocaryum aculeatum (Fig. 1). The set of genes that make up this structural rearrangement comprises ndhC, ndhK, ndhJ, trnF-GAA, and trnL-UAA.

3- In the LSC/IR and IR/SSC junctions of the plastomes, there are differences among the subtribes (Fig. 2). In Bactridinae (Acrocomia aculeata, Astrocaryum aculeatum, Astrocaryum murumuru, and Bactris gasipaes) and Elaeidinae (Elaeis guineensis) the rps19 gene is completely contained in the IR region and, therefore, there are two copies of the complete gene. In contrast, in the subtribe Attaleinae (Butia eriospatha, Cocos nucifera, and Syagrus coronata), the rps19 gene is only partially contained in the IRs, resulting in a complete rps19 and a partial rps19: the complete rps19 gene starts at IRb and ends at LSC (LSC/IRb); the partial rps19 starts at IRa but does not contain the final portion of the gene (Fig. 2).

- Similarly, the ycf1 gene is partially contained in IRs, with a complete ycf1 at IRa/SSC and a partial (pseudo) ycf1 at IRb. The ndhF gene has both position and length conserved at the IRb/SSC junction in tribe Cocoseae, with the portion of the gene contained in the IRb overlapping the ycf1 gene (56 bp) (Fig. 2).

**III-Hypervariable regions**

1-They carried out the SV% and PIS% estimates to identify the plastome regions with the greatest variation within tribe Cocoseae. All ten regions selected according to the highest SV% showed greater variation than the plastid molecular markers commonly used for phylogenetic analysis in palms (Fig. 3a). As expected, **they have SV% lower than the nuclear markers PRK and RPB2 (Fig. 3a).** Among the ten regions selected according to the highest PIS% values, all showed greater values than the plastid molecular markers commonly used for phylogenetic analysis in palms (Fig. 3b), and **two of them (trnCpetN and psbC-trnS) were more variable than the nuclear marker PRK (Fig. 3b).** The nuclear marker RPB2 showed the highest values for both SV% and PIS% estimates.

2- The substitution frequency was ~5x higher than InDels in plastomes (Table 4). In the coding sequences (CDSs), substitutions are ~80x more common than InDels. In IGSs and introns, substitutions are ~4x and ~3x more frequent than InDels, respectively (Table 4). In general, these data show that in all regions of Cocoseae plastomes, **there is a higher frequency of substitutions than InDels.** (conclusion)

**VI-*Phylogenomic analysis***

The phylogenetic tree inferred from the alignment of the ten regions with Maximum Likelihood (ML), and the greatest PIS values showed a similar or identical topology. It differed only by the presence of a polytomy in the Attaleinae clade, generated by the **low bootstrap** support in intergeneric relationships.

For the topologies generated by ML in the three datasets, the monophyly of the subtribes Bactridinae and Attaleinae was confirmed. Also, the subtribe Elaeidinae appears more closely related to Bactridinae than to Attaleinae. In Bactridinae, Bactris and Astrocaryum are closely related genera, and in Attaleinae, Cocos nucifera is sister to Syagrus coronata.

Comments: even the phylogenetic inferred from the alignment with ML and PIS value showed identical topology, and data from the LSC/IR and IR/SSC junctions and gene content of the plastomes support that subtribes Bactridinae and Elaeidinae are more related than Attaleinae, but that is not enough because they are from the same tribe Cocoseae. Authors supposed to make a comparative phylogenetic analysis with another family far in the cladistics like a phoenix.

**Comments on the study**

1-There is a considerable effort and time exhausted to execute this study.

2- The methods, results well presented.

3- This study lacks Originality and novelty.

2- The main study objective is the use of the plastidial regions and the nuclear markers PRK and RPB2 for a suitable approach for phylogenetic inferences and produces phylogenetic trees with well-resolved and the expected topologies in tribe Cocoseae. Without using another subfamily far from the Cocoseae subtribe, e.g. *P dactylifera*, the conclusion cannot be drawn with confidence.

5- This work, after a minor revision, deserves appreciation and, consequently, publication
