# Supplementary material for: The plastome sequence of Bactris gasipaes and evolutionary analysis in tribe Cocoseae (Arecaceae)
Source: PLoS One. 2021 Aug 24;16(8):e0256373. doi: 10.1371/journal.pone.0256373 (PMC8384209; doi:10.1371/journal.pone.0256373)
Supplement: Attachment — Submitted filename: Response to Reviewers.doc [file pone.0256373.s004.doc]

**Curitiba, July 31, 2021**

**Dear Dr. Berthold Heinze**

**Academic Editor of PLOS ONE**

**Manuscript ID: PONE-D-21-11791**

Manuscript resubmission Requirement

We received the results of evaluation of the refereed MS. We thank the corrections and recommendations, which improved the MS overall quality. All the queries, recommendations and suggestions were considered, as follow:

**Academic editor**

We have received three reviews for this manuscript. All authors emphasize that the study is straightforward and mostly well done. Normally, I am reluctant to recommend accepting manuscript which describes chloroplast sequencing, because this has become routine work, really (but be aware to rely too heavily on reference-guided assembly! Have a look at a book chapter where I rather recommend to publish such data as "data reports": <https://doi.org/10.1007/978-1-0716-0997-2_5>). So my recommendation is to include a Phoenix species, as some reviewers have done, and mention and discuss the study recommended (with the 100 primers for monocotyledon chloroplasts).

**R: Thank you for the positively evaluation and the constructive comments. We assembled the plastome using a *de novo* strategy. The reference-guided strategy was only used to order the contigs. The final assemble was verified by read mapping to ensure no contigs were mistakenly united.**

**We have one species from subfamily Coryphoideae (the same subfamily of *Phoenix*) in the phylogenetic analysis. Also, *Archontophoenix alexandrae* (F.Muell.) H.Wendl. & Drude from subfamily Arecoideae, but Areceae subtribe.**

**We included the recommended study (Scarcelli et al. 2011) in the discussion.**

**#Reviewer 1**

This is a straightforward but sound description of the newly sequenced plastome of the economically important palm, *Bactris gasipaes*. The properties of the plastome are analysed in a simple phylogenetic framework including seven other species from tribe Cocoseae plus a small outgroup. The analysis is fine, the manuscript is well written and the discussion makes sense. My only comment is that the authors have overlooked a paper on phylogenetically informative plastid regions in palms: https://journals.plos.org/plosone/article?id=10.1371/journal.pone.0019954 I think the discussion would benefit from a comparison of the variable regions identified here to those of Scarcelli et al.

**R: Thank you for the positively evaluation and the constructive comments. The discussion were improved and the following sentences were included: “Also, Scarcelli et al. [51] reported 100 primers for phylogeny in monocots. However, four of the hypervariable regions reported in our study were not contemplated (e.g., psaC-ndhE, petN-psbM, accD-psaI, trnS-trnG). Among them. trnS-trnG and accD-psaI primers designed by Scarcelli et al . [51] showed no amplification in Arecaceae, and petN-psbM and psaC-ndhE were not mentioned, probably due to gene rearrangements in monocots plastomes. This reinforces that the highly variable regions vary between clades, and their identification may be necessary for distinct taxonomic levels.”.**

**#Reviewer 2**

In this paper, authors sequenced and characterized the plastome of *Bactris gasipaes* (Bactridinae) and compared it with eight species from the three Cocoseae sub-tribes to perform comparative analysis and to identify hypervariable regions.

**R: Thank you for the positively evaluation.**

**#Reviewer 3**

The main study objective is the use of the plastidial regions and the nuclear markers PRK and RPB2 for a suitable approach for phylogenetic inferences and produces phylogenetic trees with well-resolved and the expected topologies in tribe Cocoseae. Without using another subfamily far from the Cocoseae subtribe, e.g. P dactylifera, the conclusion cannot be drawn with confidence.

**R: In the phylogenetic analysis, we included *Brahea brandegeei* (Purpus) H. E. Moore from the subfamily Coryphoideae (the same subfamily of *Phoenix*) and *Archontophoenix alexandrae* (F.Muell.) H.Wendl. & Drude from subfamily Arecoideae, but Areceae subtribe. These species as outgroup contemplates the suggestion. Also, we included the average depth of coverage of 67.64 (SD = 24.32).**

Best regards,

**Leila do Nascimento Vieira**

leilavieira@ufpr.br

Corresponding author
